# Hormone receptor-negative as a predictive factor for pathologic complete response to neoadjuvant therapy in breast cancer

**DOI:** 10.31744/einstein_journal/2019AO3434

**Published:** 2019-01-17

**Authors:** Luana Carolina Ferreira Fiuza Silva, Lilian Silva Martins de Arruda, Waldec Jorge David, Felipe José Silva Melo Cruz, Damila Cristina Trufelli, Auro del Giglio

**Affiliations:** 1 Programa de Pós Graduação , Faculdade de Medicina do ABC , Santo André , SP , Brazil .

**Keywords:** Breast neoplasms/pathology, Neoadjuvant therapy, Predictive value of tests, Prognosis, Survivorship (Public Health), Neoplasias da mama/patologia, Terapia neoadjuvante, Valor preditivo dos testes, Prognóstico, Sobrevivência (Saúde Pública)

## Abstract

**Objective:**

To define a predictive factor for pathologic complete response, compare the oncologic outcomes associated with the degree of pathologic response after neoadjuvant chemotherapy, and to analyze pathologic complete response as a prognostic factor for overall survival and progression-free survival.

**Methods:**

A retrospective study of patients admitted to *Hospital Estadual Mário Covas* and *Hospital Anchieta* from 2008 to 2012, with locally advanced breast cancer. Hormone receptor status, HER2 status, histologic and nuclear grade, age upon diagnosis and histological type of the tumor were analyzed. Pathologic evaluation of the tumor was subdivided into pathologic complete response, defined by the absence of tumor; intermediate response, considered as a favorable stage; and poor response, considering low-responder patients. Data obtained were submitted to statistical analysis.

**Results:**

The study included 243 patients. There was an association of pathologic complete response with HER-2 negative, histological grade 3, stage III, hormone receptor negative, positive lymph node, older age and more advanced tumors. However, after multivariate analysis the only predictor of pathologic complete response was the presence of negative hormone receptor. By analyzing the prognostic factors, hormone receptor negative was considered as an independent risk factor, and pathologic complete response was considered as an independent protective factor.

**Conclusion:**

Hormone receptor negative is predictive of pathologic complete response and is an isolated risk factor for lower progression-free survival and overall survival. Pathologic complete response is a protective factor for these same survival analyses.

## RESUMO

**Objetivo:**

Definir um fator preditivo para resposta patológica completa, comparar os resultados oncológicos associados com o grau de resposta patológica, após quimioterapia neoadjuvante, e analisar a resposta patológica completa como fator prognóstico para sobrevivência global e livre de progressão de doença.

**Métodos:**

Estudo retrospectivo de pacientes admitidas no Hospital Estadual Mário Covas e Hospital Anchieta, no período de 2008 a 2012, com câncer de mama localmente avançado. Foram utilizados *status* dos receptores hormonais, proteína HER2, grau histológico e nuclear, idade do paciente ao diagnóstico e tipo histológico do tumor. A avaliação patológica do tumor foi subdividida em resposta patológica completa, definida com ausência de tumor; resposta intermediária, considerada como um estádio favorável; e resposta ruim, considerando os pacientes pouco respondedores. As informações obtidas foram submetidas à análise estatística.

**Resultados:**

Foram incluídas 243 pacientes. Verificou-se associação de resposta patológica completa entre HER-2 negativo, grau histológico 3, estadiamento III, receptor hormonal negativo, linfonodo positivo, maior idade e tumores mais avançados. Porém, após análise multivariada, o único fator preditivo de resposta patológica completa foi presença de receptor hormonal negativo. Ao analisar fatores prognósticos, receptor hormonal negativo permaneceu como variável independente de risco, e resposta patológica completa, como variável independente de proteção.

**Conclusão:**

O receptor hormonal negativo é fator preditivo isolado de resposta patológica completa e fator de risco para menor sobrevida livre de doença e sobrevida global. Já a resposta patológica completa é fator protetor para estas mesmas análises de sobrevivência.

## INTRODUCTION

Breast cancer has been the leading cause of death in Brazilian women since 1979. Mortality rates remain high, probably because the disease is still diagnosed in advanced stages. ^(^ ^1^ ^)^ Locally advanced breast cancer is classified according to the staging system proposed by the American Joint Committee on Cancer (AJCC) as IIB, IIIA, IIIB and IIIC, 25 to 30% of which are inoperable. ^(^ ^2^ ^)^ One of the therapeutic modalities is neoadjuvant chemotherapy, which increases the likelihood of conservative surgery, also allowing initial treatment of micro metastatic disease. Furthermore, this approach allows assessing resistance to the chemotherapy regimen initially administered, offering an excellent opportunity to determine the best treatment regimen for the patient. ^(^ ^2^ ^,^ ^3^ ^)^ The MD Anderson Cancer Center has assessed more than 800 patients with disease in stages IIIA and IIIB over 25 years, and has attained objective response results in 60 to 80% of cases, complete clinical response in 15 to 20%, and pathologic complete response (pCR) in 5 to 10%, increasing the likelihood of conservative surgery. ^(^ ^4^ ^)^

Pathologic complete response is defined as the absence of residual invasive tumor in surgical tissue specimens from breast and axillary lymph nodes. ^(^ ^2^ ^,^ ^4^ ^)^ Tumor size, hormone receptor status, human epidermal growth factor receptor 2 (HER-2), molecular subtype and histological type are factors known to be associated with the pathologic response of the tumor. ^(^ ^5^ ^)^

Currently the most common molecular breast cancer is the luminal subtype, characterized as luminal A and luminal B. Luminal A accounts for approximately 60% of breast carcinomas and has the best prognosis when compared to other breast carcinomas. Most of them are estrogen receptor positive and low histologic grade. ^(^ ^6^ ^)^ Luminal B is characterized by expressing genes associated with HER2, and by more cell proliferation genes, including the expression of MKi67 (Ki-67), CCNB1 and MYBL2 genes. Its higher cell proliferation rate is related to worse prognosis when compared to luminal A. ^(^ ^7^ ^)^ Overexpression of HER2 occurs in 10 to 15% of breast cancers, is frequently hormone-receptor-negative, and has the second worst prognosis when compared to patients who do not show this gene amplification, although molecularly targeted therapy improves the prognosis.

The triple negative clinical phenotype is negative for hormonal receptors and for HER2 overexpression. It mainly comprises the basal-like molecular subtype and shows substantial heterogeneity. In a study of DNA and RNA utilization, four stable subtypes were identified: luminal/androgen receptor, mesenchymal, basal-like immune suppressed, and basal-like immune activated. ^(^ ^8^ ^)^ These four subtypes have the worst prognosis, with lower progression-free survival and overall survival, which can be attributed to their biological characteristics. ^(^ ^9^ ^)^ The triple-negative breast cancer proliferation rate and BRCAness are two characteristics that illustrate these facts. ^(^ ^10^ ^)^

Anthracycline and taxane regimen-based chemotherapy has increased the rate of complete pathologic response. ^(^ ^5^ ^,^ ^11^ ^)^ In contrast, the pCR rate is low in the luminal A molecular subtype, reaching only 6.7%. ^(^ ^11^ ^-^ ^14^ ^)^

The role of persistent *in situ* lesion is controversial. A meta-analysis from a German group demonstrated that persistence of a residual *in situ* lesion is associated with shorter disease-free survival (DFS) when compared to pCR (absence of invasive component and *in situ* component). ^(^ ^15^ ^)^

## OBJECTIVE

To define the predictive factors for pathologic complete response, as well as to analyze its role as a prognostic factor for overall survival and progression-free survival.

## METHODS

A retrospective study including patients aged over 18 years, with invasive breast carcinoma confirmed by pathology examination, locally advanced (stages II and III, according to AJCC), performance status between zero and 2, and without previous oncological therapy, treated with neoadjuvant chemotherapy followed by radical or conservative surgical resection.

We reviewed the medical records of the hospitals linked to the *Faculdade de Medicina do ABC* (FMABC), Santo André (SP), from 2008 to 2012. Patients with distant metastasis upon diagnosis and/or with a second primary tumor were excluded. The study was approved by the Research Ethics Committee of FMABC, opinion 394.576, CAAE: 20730713.2.0000.0082.

We used hormone receptor status, HER2 protein status, histological and nuclear grade, patient age at diagnosis, lymph node presence, and clinical and pathological tumor staging. Estrogen and progesterone receptors were considered positive if ≥10% of the stained positive or if Remmele score ≥3, ^(^ ^16^ ^)^ taking into consideration frequency and intensity of staining. Human epidermal growth factor receptor 2 protein status was assessed by immunohistochemistry, and considered positive if the score was equal to 3, or fluorescence *in situ* hybridization (FISH) positive. Histologic and nuclear grades were used to describe cell proliferation. The pathology assessment of the tumor after resection was subdivided into three groups: pathologic complete response defined by absence of tumor (ypT0ypN0), intermediate response considered as favorable stage (ypT1-2ypN0) and poor response considering low-responder patients (ypT3-4ypN1-3). Among histopathological factors with prognostic value, tumor size and lymph node involvement are variables that have a greater impact on the definition of individual risk, ^(^ ^17^ ^)^ and also on the 5 to 20-year distant recurrence rates, which for T1N0 patients is 14%, for T2N0 is 21%, and rates range from 23% to 47% for TxN+.

The treatments included neoadjuvant chemotherapy with doxorubicin, cyclophosphamide, paclitaxel (AC-T); dose-dense AC-T; or other chemotherapy regimens, associated or not to trastuzumab (in HER2 positive tumors), followed by surgery, which included radical mastectomy, lumpectomy or quadrantectomy.

### Statistical analysis

Continuous variables were described using means and standard deviation, or medians and minimum and maximum values. Categorical variables were described by absolute and relative frequencies. Student’s *t* test was used to compare the means of two sample populations, and ANOVA with Bonferroni’s auxiliary test was used for the comparison among the means of three or more populations. Comparisons of the frequency of a phenomenon between groups of categorical variables were performed using Fisher’s exact test and χ ^2^ test. Multivariate analysis to determine the predictive factors of pCR was performed by logistic regression, and all variables that presented p<0.2 in the univariate analysis were tested. The Kaplan-Meier method was used for progression-free survival and overall survival analyses. Patients were censored on the date of the event (death and/or progression) or most recent contact. Curves were compared using the log-rank test. For both multivariate analysis and univariate analysis, the Cox regression model was used to determine prognostic factors for calculating hazard ratio (HR), 95% confidence interval (95%CI), and p≤0.2 value was considered for all variables. All analyses were performed using the Statistical Package for the Social Sciences (SPSS), version 17.0 (SPSS ^®^ Inc., Illinois, USA). An alpha or type I error with a value ≤5% (p<0.05). was established for all comparisons.

## RESULTS

A total of 243 patients were identified with locally advanced invasive breast cancer and submitted to neoadjuvant chemotherapy. The median age of patients was 52 years, ranging from 27 to 87 years. Clinical and pathological characteristics are described on tables 1 and 2 , respectively.

**Table 1:** Descriptive analysis of the clinical characteristics of all patients included

Characteristics	n (%) ^†^
Age, years	
<65	199 (81.9)
>65	44 (18.1)
Median	52.0±13 ^*^
Surgery	
Quadrantectomy	50 (20.6)
Lumpectomy	9 (3.7)
Mastectomy	184 (75.7)
Clinicla staging	
II	74 (30.5)
III	169 (69.5)
Tumor	
T2	44 (18.1)
T3	122 (50.2)
T4	76 (31.3)
Lymph node	
N0	69 (28.4)
N+	174 (71.6)
Neoadjuvant CT regimen	
AC-T	174 (71.6)
AC-T dose-dense	20 (8.2)
Others	49 (20.2)
Herceptin	
Yes	47 (19.3)
No	196 (80.7)
Disease progression	
Yes	171 (70.4)
No	72 (29.6)
Local recurrence	
Yes	32 (13.2)
No	211 (86.8)
Systemic recurrence	
No	185 (76.1)
Bones	22 (9.1)
Lung	19 (7.8)
Central nervous system	9 (3.7)
Liver	4 (1.6)
Others	4 (1.6)
Death	
No	206 (84.8)
Yes	37 (15.2)

^*^ mean±standard deviation, in years; ^†^ total number of patients included, not necessarily corresponding to the sum of items of each variable, due to lack of information in the charts. T2: tumor >2cm and ≤5cm; T3: tumor >5cm; T4: tumor of any size, with direct extension to the chest wall, skin or both, or inflammatory tumor; N0: absence of lymph node involvement; N+: presence of lymph node involvement; CT: chemotherapy; AC-T: doxorubicin, cyclophosphamide, paclitaxel.

**Table 2:** Pathological characteristics of all patients

Characteristics	Total n (%)
Histological type	
Invasive ductal	217 (89.3)
Invasive lobular	12 (4.9)
Others	14 (5.8)
Histological grade	
I	13 (5.4)
II	155 (64.0)
III	74 (30.6)
Nuclear grade	
I	4 (1.6)
II	118 (48.6)
III	121 (49.8)
Hormone receptor	
Positive	150 (61.7)
Negative	93 (38.3)
HER2 protein	
Positive	58 (23.9)
Negative	185 (76.1)
Pathological response	
Pathological complete response	75 (30.9)
Intermediate response	50 (20.6)
Poor response	118 (48.6)
Tumor (ypT)	
ypT0	80 (32.9)
ypT1	32 (13.2)
ypT2	66 (27.2)
ypT3	45 (18.5)
ypT4	20 (8.2)
Lymph node (ypN)	
ypN0	141 (58.0)
ypN+	102 (42.0)

HER-2: human epidermal growth factor receptor 2.

**Figure 1:**
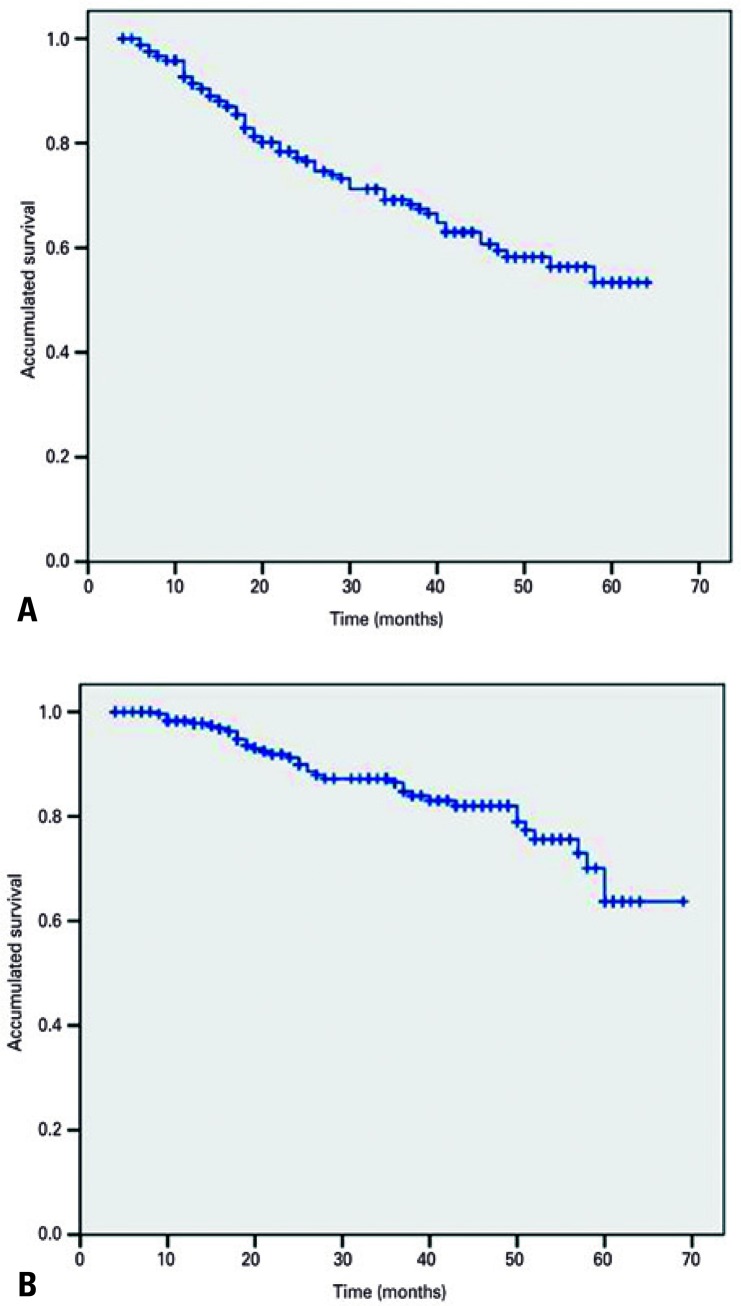
Kaplan-Meier survival curves. (A) Disease-free survival and (B) Overall survival

The median follow-up of patients was 32 months, ranging from 4 to 69 months. Overall survival and progression-free survival did not reach the median (63.7% and 53.4%, respectively), and are shown in figure 1 .

### Predictive factors of pathologic complete response

In order to assess the predictive factors of pCR, associations were made among all variables. The univariate analysis showed that pCR was associated with HER2 negative, histologic grade 3, stage III, negative hormone receptor, positive lymph node, older age and more advanced tumors (T3/T4). However, after the multivariate analysis, the only predictive factor of pCR was the presence of negative hormone receptor (HR = 2.2; 95%CI: 1.25-3.89; p=0.006, logistic regression).

### Pathologic complete response as a prognostic factor

**Figure 2:**
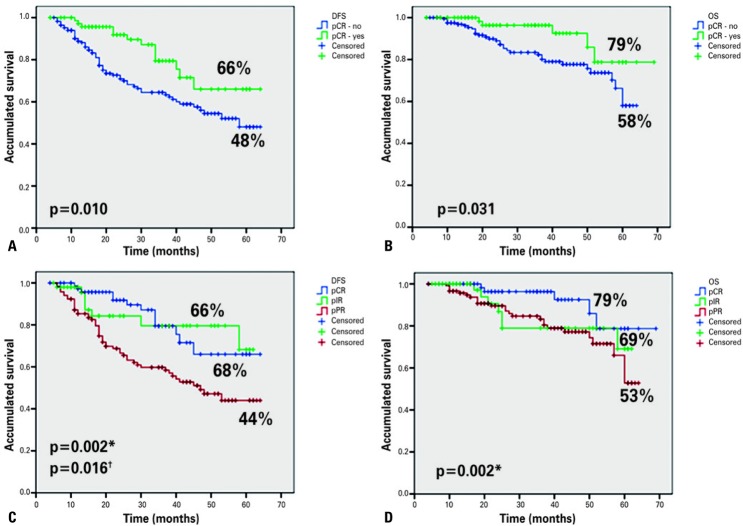
Comparison among the types of pathological response. (A) Disease-free survival. (pCR): yes or not. (B) Overall survival. pCR: yes or not. (C) Disease-free survival. pCR, pIR and pPR. (D) Overall survival. pCR, pPIR and pPR

The accumulated DFS for pathologic complete response, intermediate response and poor response was 66%, 68% and 44%, respectively; and for overall survival, 78%, 69% and 52%, respectively. In order to better analyze the results, we divided the patients into two groups: pCR, yes or no. Thus, the following results were found: patients with pCR had overall survival of 78% *versus* 58%, and DFS of 66% *versus* 48%, as compared to those with intermediate or poor response ( Figure 2 ).

* p value refers to comparison of pCR with pPR; ^†^ p value refers to comparison of pathological intermediate response with pPR.

DFS: disease-free survival; pCR: pathological complete response; pIR: pathological intermediate response; pPR: pathological poor response; OS: overall survival.

In addition to pCR, in the univariate analysis we observed that the variables related to DFS were nuclear grade, hormone receptor and clinical tumor staging. In the multivariate analysis, we found that the only independent risk factors were negative hormone receptor for shorter DFS and pCR for longer DFS. For overall survival, the following variables were included in the multivariate analysis: nuclear grade, hormone receptor and pCR, and hormone receptor negative also remaining as an independent risk variable, and pathologic complete response as independent protection variable, as shown in tables 3 and 4 .

**Table 3:** Univariate and multivariate analysis for disease-free survival

Characteristics	Univariate analysis			Multivariate analysis		
	
	HR	95%CI	p value	HR	95%CI	p value
Age	1.0	0.99-1.03	0.302		N/A	
Clinical staging	1.66	0.94-2.93	0.081	1.6	0.89-2.79	0.12
Tumor	1.94	0.93-4.04	0.078	1.4	0.57-3.45	0.45
Positive lymph node	1.12	0.66-1.20	0.665		N/A	
Grade						
Histological	1.26	0.78-2.03	0.344	1.18	N/A	0.507
Nuclear	1.49	0.90-2.32	0.124		0.721-1.95	
HER2 status	1.34	0.88-2.24	0.266		N/A	
Hormone receptor	1.76	1.14-2.80	0.016	3.38	1.73-6.60	<0.001
pCR	0.46	0.25-0.85	0.013	0.26	0.09-0.66	0.005

p value: probability of obtaining results, or something more extreme, if the null hypothesis is true. HR: hazard ratio; 95%CI: 95% confidence interval; N/A: not applicable; pCR: pathological complete response.

**Table 4:** Univariate and multivariate analysis for overall survival

Characteristics	Univariate analysis			Multivariate analysis		
	
	HR	95%CI	p value	HR	95%CI	p value
Age	1.17	0.52-2.68	0.706		N/A	
Clinical staging	1.24	0.58-2.63	0.580		N/A	
Tumor	1.08	0.45-2.06	0.860		N/A	
Positive lymph node	1.40	0.63-3.04	0.410		N/A	
Grade						
Histological	1.50	0.78-2.89	0.220		N/A	
Nuclear	2.09	1.03-4.23	0.041	1.6	0.76-3.33	0.216
HER2 status	0.72	0.32-1.63	0.420		N/A	
Hormone receptor	2.50	1.30-4.84	0.006	2.20	1.37-3.55	0.001
pCR	0.37	0.14-0.95	0.038	0.37	0.19-0.68	0.002

p value: probability of obtaining results, or something more extreme, if the null hypothesis is true. HR: hazard ratio; 95%CI: 95% confidence interval; N/A: not applicable; pCR: pathological complete response.

## DISCUSSION

Neoadjuvant chemotherapy is the initial standard treatment for locally advanced breast cancer. The correlation between the response to neoadjuvant chemotherapy and prognostic factors allows us to understand that the different subtypes of breast cancer have differentiated response profiles. Pathologic complete response is a predictor of long-term outcome and is therefore an appropriate marker for survival, although the incidence and prognostic impact of pCR vary among breast cancer subtypes. ^(^ ^4^ ^,^ ^15^ ^,^ ^18^ ^)^ The pCR rate increases in triple-negative and HER2 positive tumors (28 to 32%), according to the prospective and randomized NOAH study, which showed significantly higher event-free survival in patients treated with chemotherapy and trastuzumab (71% *versus* 56%, p=0.013) and also a significantly higher pCR rate (43% *versus* 23%, p=0.002), ^(^ ^19^ ^)^ which agrees with the series reported here. The ACOSOG Z1041 study ^(^ ^20^ ^)^ compared 4 cycles of FEC75, followed by 12 weeks of paclitaxel (80mg/m ^2^ ) concomitant with trastuzumab, with the experimental arm consisting of 12 weeks of paclitaxel, followed by 4 cycles of FEC75, concomitant with trastuzumab throughout the treatment. Pathological complete response rates found were high in both arms, although in the subgroup of patients with hormone receptor negative, the pCR was higher than in patients with hormone receptor positive.

In the present study, with data from two public reference hospitals in oncology, with a high percentage of cases with locally advanced disease, data obtained were similar to those in the literature. The multivariate analysis showed that the only predictive factor for pCR was the presence of negative hormone receptor. Hormone receptor negative tumors tend to have a higher rate of pathologic response to chemotherapy than hormone receptor positive tumors. ^(^ ^21^ ^)^ An important neoadjuvant study, the GeparSixto of the German AGO-B and GBG groups, also demonstrated high complete pathology response rate in triple-negative tumors. ^(^ ^22^ ^)^

In another analysis of pathologic complete response as a prognostic factor, the only independent risk factor for shorter DFS and overall survival was hormone receptor negative, and pCR was the only protective factor for these same survival analyses. Patients with hormone receptor negative who achieve pCR present a prognosis comparable to luminal A tumors. ^(^ ^14^ ^)^ Similarly, patients with triple negative neoplasms presenting pCR have a better prognosis when compared to those with residual disease after neoadjuvance. ^(^ ^23^ ^)^ According to a study at MD Anderson analyzing post-mastectomy pathology specimens of 241 patients treated with neoadjuvant paclitaxel followed by FAC, and of 141 treated with neoadjuvant FAC, patients with extensive residual disease were observed to have a worse prognosis, regardless of hormone receptor status, adjuvant hormone therapy, or pathological stage of residual disease, according to the AJCC. ^(^ ^24^ ^)^

The NSABP B18 study, from the National Surgical Adjuvant Breast and Bowel Project (NSABP), demonstrated increased overall survival and disease-free survival in patients who presented pCR compared with those who did not. ^(^ ^25^ ^)^

Some studies have shown the association between pCR and the cell proliferation index measured by Ki-67. ^(^ ^26^ ^-^ ^28^ ^)^ One of the limitations of our study was the unavailability of Ki-67 to measure cell proliferation as a predictor variable, due to the lack of registration of this examination in many patient records.

## CONCLUSION

The presence of negative hormonal receptor was an isolated predictive factor of pathologic complete response and was associated with shorter overall survival and disease-free survival. Pathologic complete response was associated with longer disease-free survival and overall survival.
